# Synthesis, Characterization, and Bioactivity of Mesoporous Bioactive Glass Codoped with Zinc and Silver

**DOI:** 10.3390/ijms241813679

**Published:** 2023-09-05

**Authors:** Tsung-Ying Yang, Guann-In Chern, Wei-Hsun Wang, Chi-Jen Shih

**Affiliations:** 1 Department of Medical Laboratory Science, I-Shou University, Kaohsiung 84001, Taiwan; zegma040899@gmail.com; 2 Research Organization for Nano and Life Innovation, Future Innovation Institute, Waseda University, Tokyo 162-0041, Japan; 3 Research Institute for Science and Engineering, Waseda University, Tokyo 169-8555, Japan; 4 School of Education, Waseda University, Tokyo 169-8050, Japan; 5 Department of Fragrance and Cosmetic Science, College of Pharmacy, Kaohsiung Medical University, Kaohsiung 80708, Taiwan; style9720428@gmail.com; 6 Department of Orthopedic Surgery, Changhua Christian Hospital, Changhua 50006, Taiwan; 7 Department of Post-Baccalaureate Medicine, College of Medicine, National Chung Hsing University, Taichung 40227, Taiwan; 8 School of Medicine, College of Medicine, Kaohsiung Medical University, Kaohsiung 80708, Taiwan; 9 Department of Chemical Engineering, National United University, Miaoli 36063, Taiwan; 10 Department of Golden-Ager Industry Management, Chaoyang University of Technology, Taichung 41349, Taiwan; 11 Department of Medical Imaging and Radiology, Shu-Zen Junior College of Medicine and Management, Kaohsiung 82144, Taiwan; 12 Drug Development and Value Creation Research Center, Kaohsiung Medical University, Kaohsiung 80708, Taiwan; 13 Department of Medical Research, Kaohsiung Medical University Hospital, Kaohsiung 80708, Taiwan

**Keywords:** silver, zinc, biomaterial, osteoinduction, antibacterial, drug-resistant bacteria

## Abstract

Due to the overconsumption of antimicrobials, antibiotic-resistant bacteria have become a critical health issue worldwide, especially methicillin-resistant *S. aureus* (MRSA) and vancomycin-resistant *E. faecalis* (VRE). Recently, many efforts have been made to load metals into bioactive glasses to enhance the multifunctionality of materials, such as antibacterial and osteoinductive functions. Zinc has been documented to stimulate the gene expression of various regulatory factors in bone cells. Meanwhile, previous studies have reported that silver and zinc could be a promising antibacterial combination with synergistic antimicrobial effects. Here, we sought to develop a biomaterial coreleasing zinc and silver, designated 80S-ZnAg, and to evaluate its antibacterial activity and biocompatibility. The textural analyses demonstrated different coreleasing patterns of zinc and silver for the materials. The chemical characterization revealed that the zinc in 80S-ZnAg could be the network modifier when its molar ratio was high, releasing more zinc; zinc could also be the network former when its molar ratio was low, showing an extremely low rate of release. However, the ICP results for 80S-Zn3Ag2 demonstrated up to 7.5 ppm of zinc and 67.6 ppm of silver. Among all the 80S-ZnAg materials, 80S-Zn3Ag2 demonstrated more marked antibacterial activity against MRSA and VRE than the others, with inhibition zones of 11.5 and 13.4 mm, respectively. The cytotoxicity assay exhibited nearly 90% cell viability at 20 mg/mL of 80-Zn3Ag2. Further clinical study is needed to develop an innovative biomaterial to address the issue of antibiotic resistance.

## 1. Introduction

Bone-related infectious diseases, such as osteomyelitis and periarthritis, are mostly caused by surgical/accidental injuries accompanied by bacterial infections [1,2]. Previous studies have reported that up to 80% of osteomyelitis cases were caused by *Staphylococcus aureus* [3,4]. Previously, the commonly used treatment for *S. aureus* infections was methicillin, but due to overconsumption, the emergence of methicillin-resistant *S. aureus* (MRSA) has become a critical health issue in recent decades [5]. To address the issue of methicillin resistance, vancomycin has been used as a last-resort antibiotic for MRSA infections. However, a few instances of vancomycin-intermediate *S. aureus* have been reported [4,6], indicating the problem of antibiotic resistance. Caries, if left untreated, may erode the pulp and cause pulpitis. In serious cases, the infected pulp cavity needs to be removed and subsequently sealed in a procedure called root canal therapy. During the process, the root apex of the tooth can become prone to infection due to inappropriate sterilization or treatment, and *Enterococcus faecalis* has been named as the main culprit of postoperative infection [7]. Clinically, calcium hydroxide (Ca(OH)_2_), as a pulp-coating material, has bactericidal ability against microorganisms due to its high alkalinity, but its antibacterial effect against *E. faecalis* is poor [8]. Additionally, the high alkalinity of Ca(OH)_2_ often causes cell necrosis in the dental pulp and its surrounding regions [9]. Thus, MTA (Portland cement, Bi_2_O_3_, and CaSO_4_) and biodentine have been developed as alternatives. Owing to their lack of or low antibacterial activities, MTA and biodentine are usually combined with antibiotics, leading to more issues of antibiotic resistance [10], such as vancomycin-resistant *E. faecalis* (VRE) [11].

Bioactive glass (BG) is a widely used material that can exchange ions on its surface and generate biomimetic crystals. BG can raise pH levels in bodily fluids, creating a mildly bactericidal environment [12], which is valuable for dental and bone repair. Biomimetic crystals are materials imitating natural substances, such as hydroxyapatite (HA), which mimics the composition of bone and teeth and has osteoinductive properties [13]. HA, which is composed of calcium, phosphorus, and hydroxyl groups, is biocompatible and is valuable in bone tissue engineering due to its capacity for osteoconduction [14]. The synthesis of BG involves two methods: melt quenching and sol-gels [14]. The type of BG known as 45S5 Bioglass^®^, with 46.1% SiO_2_, 24.4% Na_2_O, 26.9% CaO, and 2.6% P_2_O_5_, is osteoinductive, osteoconductive, and biodegradable [15]. Sol-gels yield higher surface area and porosity [16]. Zhao and Vallet-Regí et al. [17,18] developed mesoporous bioactive glasses (MBGs) using sol-gels with a high surface area and bone compatibility [19]. Chiang et al. [20] and Lalzawmliana et al. [21] found MBGs to be effective in sealing dentin and bone regeneration. MBGs’ high surface area also aids as a drug carrier [22]. Zhu et al. [23] synthesized an MBG in 2016, showing promise for orthopaedic and dental restoration due to its formation of apatite and capabilities for drug release.

Recently, many efforts have been made to load metals into bioactive glasses, such as Ag^+^, Cu2^+^, Zn^2+^, and Sr^2+^, to enhance the multifunctionality of materials [24,25,26,27,28]. In those studies, the structure of the glass may be affected by the metal and further influence the ion release or formation of hydroxyapatite (HA). Among the metal ions, zinc (Zn) is indispensable in the human body. Zinc can stimulate the gene expression of various regulatory factors in bone cells, such as the activation of Runx2/Cbfa1 (the transcription factor for osteoblasts’ differentiation), the proliferation of Type I collagen, and the activation of osteocalcin and alkaline phosphatase [19,29], enhancing bone formation. Zinc can also inhibit RANKL and M-CSF in the osteoclasts, impeding the resorption and destruction of bone [30]. Naruphontjirakul et al. reported multifunctional zinc and silver codoped bioactive glass nanoparticles for bone therapy and regeneration in 2023 [31]. In addition, zinc regulates inflammation by inhibiting the secretion of NF-κB and proinflammatory factors (TNF-α, IL-1β, IL-6, and IL-8) [32,33]. Silver (Ag) is a well-known antibacterial agent and has been widely used clinically [34], including in sutures and gauze. A previous study described that nanoscale silver could release silver ions (Ag^+^) to eliminate bacteria through disrupting the cell wall and membrane, denaturating the ribosomes and proteins, blockading ATP synthesis, interrupting the electron transport chain, generating reactive oxygen species, and interfering with DNA replication [35]. Li et al. described that mesoporous bioactive glass codoped with copper and silver could promote angiogenesis through the copper ions [36].

Garza-Cervantes et al. examined the synergistic antimicrobial effects of silver and transition metals (Ni, Co, Cd, Cu, and Zn) [37]. Individual transition metal salts (NiSO_4_·6H_2_O, CoCl_2_·6H_2_O, 3CdSO_4_·8H_2_O, CuSO_4_·5H_2_O, and ZnSO_4_·7H_2_O) were combined with silver nitrate, and their antibacterial activities against *Escherichia coli* ATCC 11229 and *Bacillus subtilis* ATCC 23857 were evaluated. Synergistic effects were observed for all combinations against *E. coli* ATCC 11229 and for Zn+Ag against *B. subtilis* ATCC 23857. Although the study was conducted using individual transition metal salts, silver and zinc could be a promising antibacterial combination. In another study, Bednář et al. synthesized a silicate net nanostructure with a zinc content of up to 30% weight, and silver nitrate was then doped into the structure using a photocatalytic reduction method [38]. The nanocomposite without silver, ZnO·*m*SiO_2_, had antibacterial activities against *E. coli*, *Pseudomonas aeruginosa*, *Streptococcus salivarius*, and *S. aureus*, with MICs ranging from 10.6 to 26.5 mg/mL. Compared with ZnO·*m*SiO_2_, silver-doped ZnO·*m*SiO_2_ was observed to have more robust activities, with lower MICs ranging from 2.9 to 5.9 mg/mL. However, the study did not report the silver and zinc contents in the solutions, and the bacterial strains they evaluated were drug-sensitive. In a separate study, Azizabadi et al. prepared a senary SiO_2_-P_2_O_5_-CaO-SrO-Ag_2_O-ZnO glass system via the sol-gel method, designated BG-AZ [39]. The antibacterial results of their materials demonstrated only 80–90% bacterial eradication, which could not be defined as an MIC or an MBC. An extremely low zinc release (0.06–0.16 ppm) was noted, implying that the zinc could barely be released from the system after more than 20 days.

Previous research has underscored several essential attributes of bioactive glass, such as its ability to facilitate bone growth and serve as a drug carrier. While the incorporation of Zn^2+^ holds the potential for enhancing bone regeneration, earlier investigations examined the impact of Zn^2+^ on the crystal structure of the bioglass matrix, revealing a compromise in its antibacterial effectiveness [39]. Considering this, the introduction of silver emerges as a powerful antibacterial solution that could restore the material’s antibacterial efficacy while preserving the glass’s innate bioactivity. Here, we sought to develop a novel antibacterial biomaterial for the fields of orthopaedics and dentistry. A SiO_2_-CaO-P_2_O_5_ system coreleasing zinc and silver was synthesized, designated 80S-ZnAg, which had synergistic antibacterial effects against antibiotic-resistant bacteria, especially MRSA and VRE. The biocompatibility of the 80S-ZnAg materials was also evaluated, including their cytotoxicity and ability to form HA.

## 2. Results

### 2.1. Characterization of 80S-ZnAg

**Figure 1:**
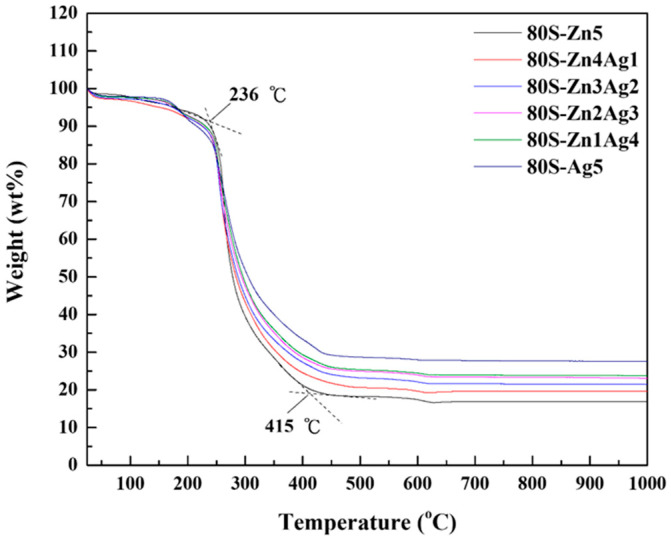
TGA curves of 80S-Zn5, 80S-Zn4Ag1, 80S-Zn3Ag2, 80S-Zn2Ag3, 80S-Zn1Ag4, and 80S-Ag5.

The thermogravimetric analysis of all materials revealed a <10% weight loss at temperatures under 236 °C, where the ethanol and water were totally removed (Figure 1). The structure-directing agent, the template, and the acid groups were eliminated at temperatures between 236 and 415 °C (the dashed lines of Figure 1), causing a weight loss of approximately 70%. No observable weight loss occurred at temperatures higher than 450 °C, whereas the thermal treatment of the material prepared in this work was 700 °C.

**Figure 2:**
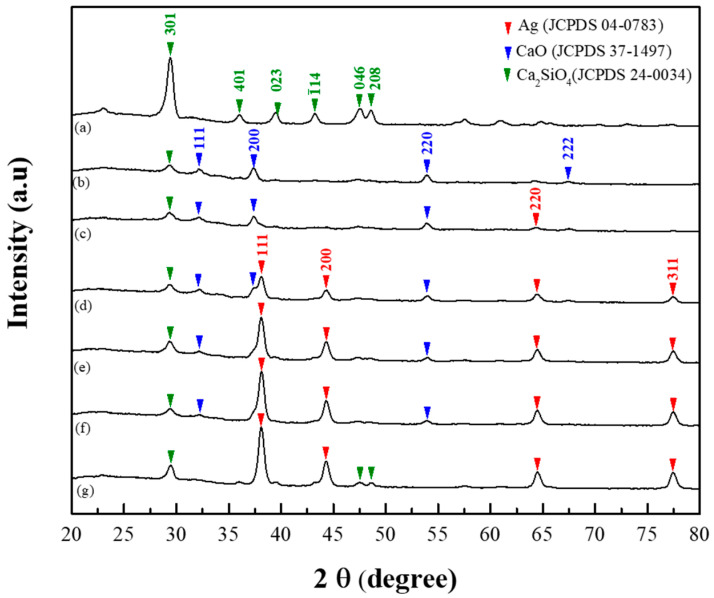
XRD patterns of (**a**) 80S, (**b**) 80S-Zn5, (**c**) 80S-Zn4Ag1, (**d**) 80S-Zn3Ag2, (**e**) 80S-Zn2Ag3, (**f**) 80S-Zn1Ag4, and (**g**) 80S-Ag5.

The crystal phases of the 80S-ZnAg materials were obtained and analysed using Jade 6 software on the basis of the JCPDS database (Figure 2). The 80S material was analysed as the control without the addition of metal, illustrating a lattice plane of (301) at 2θ = 29.35° (JCPDS No. 24-0034, calcium silicate) (Figure 2a). The same plane of calcium silicate was also observed in the metal-containing groups (Figure 2b–g). In the silver-containing materials, the lattice planes of (111), (200), (220), and (311) at 2θ = 38.11°, 44.27°, 64.42°, and 77.47°, respectively, represented the crystallinity of silver (JCPDS No. 04-0783), with an increasing trend in a dose-dependent manner (Figure 2c–g). However, the (111), (200), (220), and (222) planes at 2θ = 32.20°, 37.34°, 53.85°, and 67.37°, respectively, were found for all zinc-containing materials, representing the crystal phase of CaO (JCPDS No. 37-1497).

**Figure 3:**
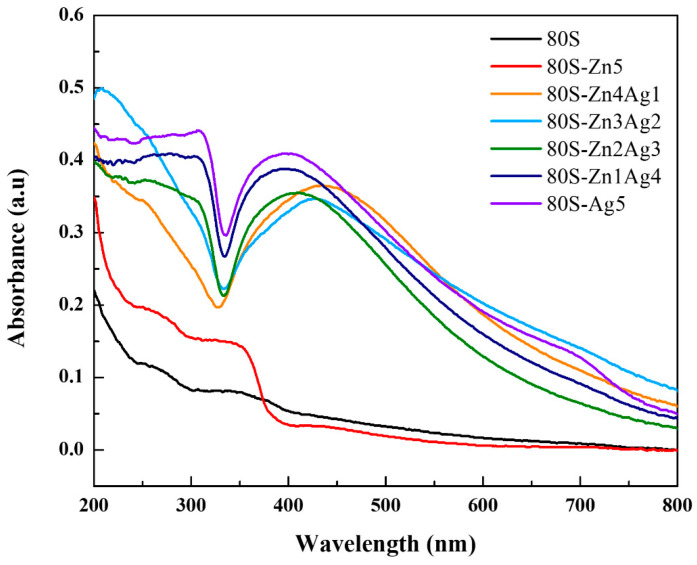
UV-vis results of 80S, 80S-Zn5, 80S-Zn4Ag1, 80S-Zn3Ag2, 80S-Zn2Ag3, 80S-Zn1Ag4, and 80S-Ag5.

The UV-vis spectra of the 80S-ZnAg materials are shown in Figure 3. The absorbance at approximately 400 nm indicated silver nanoparticles, with higher signals when the silver content increased. The result of 80S-Zn5 demonstrated a peak at 350 nm, which indicated the absorbance of ZnO.

**Figure 4:**
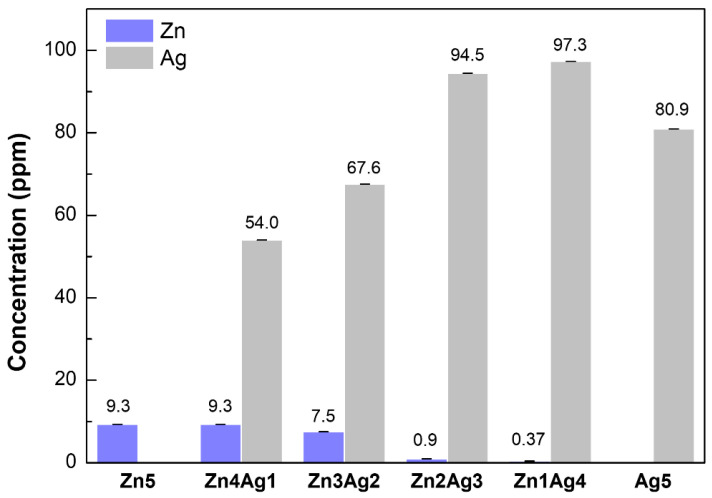
ICP-MS results of bioinspired 80S-ZnAg materials (40 mg/mL in TSB). The bars and scales represent the mean ± SD of detection in triplicate.

The silver and zinc contents in the novel 80S-ZnAg materials were then determined (Figure 4). The zinc contents were 9.3 ± 0.010, 9.3 ± 0.001, and 7.5 ± 0.006 ppm for 80S-Zn5, 80S-Zn4Ag1, and 80S-Zn3Ag2, respectively, whereas extremely low zinc contents were noted for 80S-Zn2Ag3 and 80S-Zn1Ag4, with zinc concentrations of 0.97 ± 0.014 and 0.37 ± 0.014 ppm, respectively. The silver content of 80S-Ag5 in TSB was 80.9 ± 0.012 ppm. With the addition of zinc and the decrease in the silver molar ratio, the silver contents of the bioinspired 80S-Zn4Ag1, 80S-Zn3Ag2, 80S-Zn2Ag3, and 80S-Zn1Ag4 were 54.0 ± 0.008, 67.6 ± 0.009, 94.5 ± 0.015, and 97.3 ± 0.011 ppm, respectively. The low release of zinc might be because the zinc ions form tetrahedral ZnO_4_^2−^ species as a glass former, whereas calcium is utilized to balance the charge [40]. Taken together, these results show the observable corelease of silver and zinc in the 80S-Zn4Ag1 and 80S-Zn3Ag2 preparations, but not in the others, where the material 80S-Zn3Ag2 had a higher silver content than 80S-Zn4Ag1 and a comparable zinc concentration and was chosen as the candidate for the following analyses. Furthermore, the higher silver contents of 80S-Zn3Ag2 compared with those of 80S-Ag2 after 8 h were revealed in the time-dependent release curve (Figure S1), suggesting the improved ability of 80S-Zn3Ag2 to release silver.

### 2.2. Chemical Characterization of 80S-Zn3Ag2

**Figure 5:**
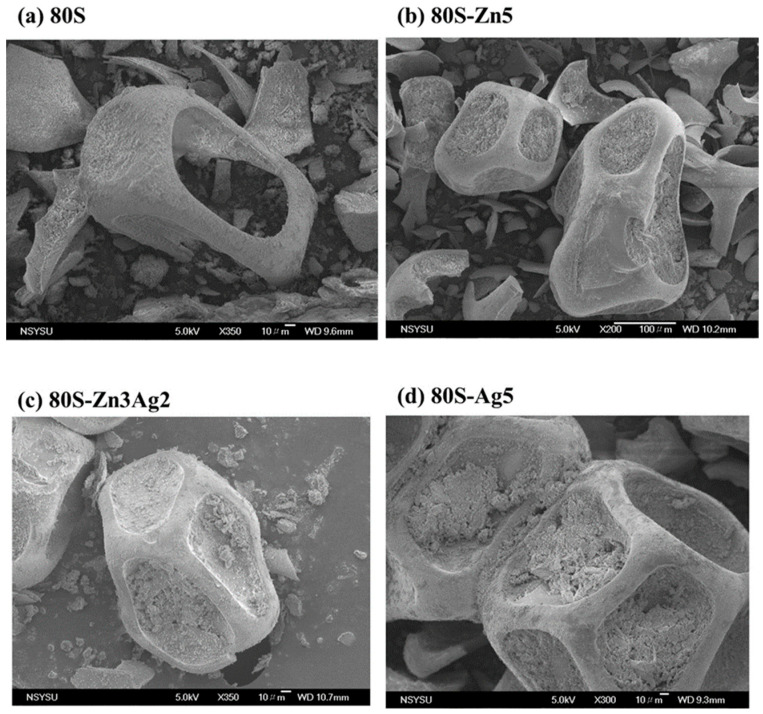
SEM micrographs of (**a**) 80S, (**b**) 80S-Zn5, (**c**) 80S-Zn3Ag2, and (**d**) 80S-Ag5.

**Figure 6:**
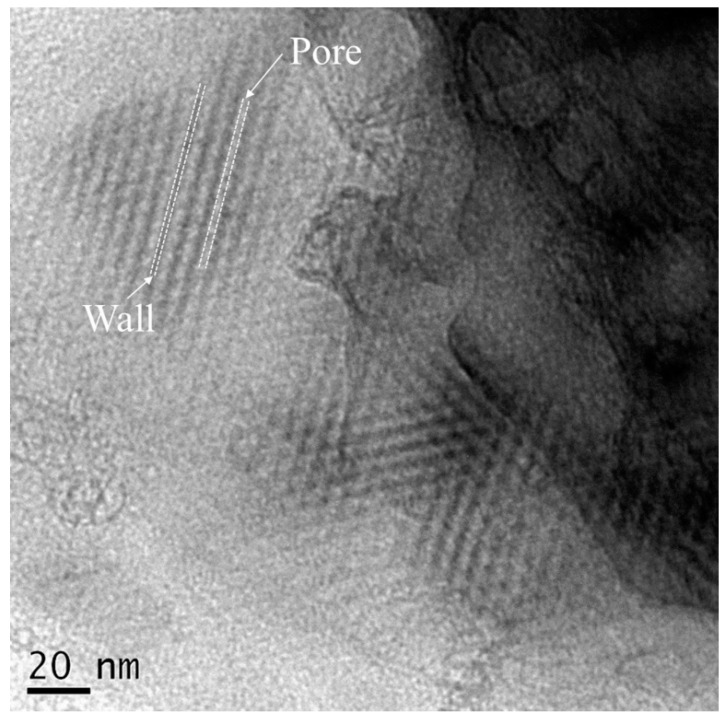
TEM micrograph of 80S-Zn3Ag2. The white dotted line represents the borders of the pores or walls.

**Figure 7:**
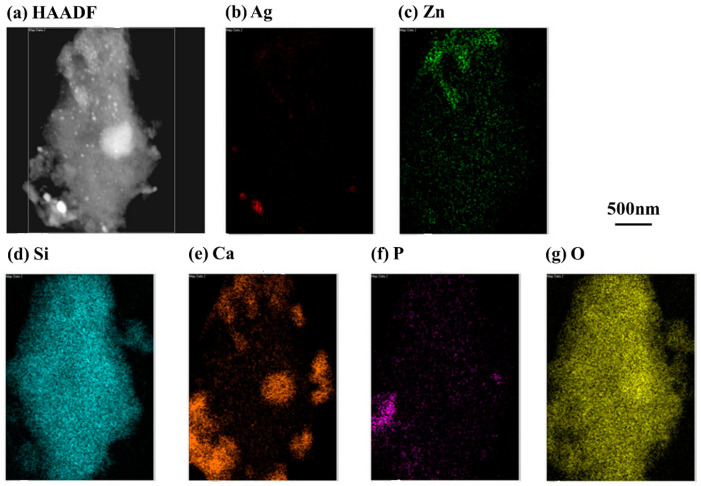
HAADF-STEM and EDS elemental mapping images of 80S-Zn3Ag2 revealed (**a**) the high-angle annular darkfield morphology and the distributions of (**b**) Ag, (**c**) Zn, (**d**) Si, (**e**) Ca, (**f**) P, and (**g**) O.

Surface images of 80S, 80S-Zn5, 80S-Ag5, and 80S-Zn3Ag2 were captured using SEM (Figure 5), and due to the low release of zinc, the elemental distributions were revealed by EDS for further validation of the textural analysis (Figure S2). The PUF template used for the preparation of the materials sculpted macropores up to 150 μm in all materials tested (Figure 5). The EDS elemental analyses illustrated that silver and zinc were successfully incorporated into the glass structure (Figure S2). TEM micrographs revealed the walls and pores of 80S-Zn3Ag2, indicating that 80S-Zn3Ag2 was a mesoporous glass (Figure 6). HAADF-STEM with EDS analysis produced elemental mapping images of 80S-Zn3Ag2 (Figure 7). Silver and calcium were more distributed in the bright white regions (Figure 7a,b,e), whereas zinc was distributed evenly (Figure 7c), similar to Si, P, and O (Figure 7d,f,g), suggesting the different roles of silver and zinc in the material.

**Figure 8:**
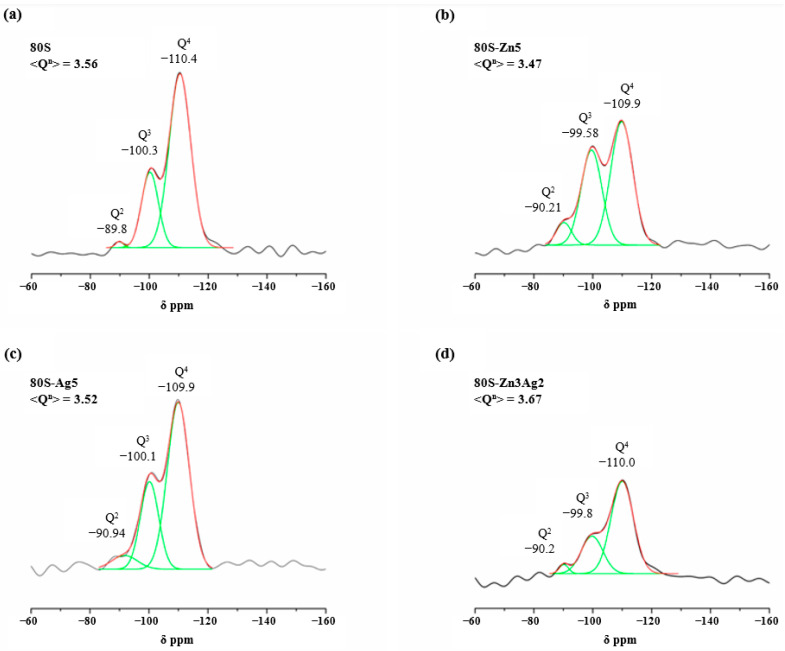
Solid-state ^29^Si MAS NMR of (**a**) 80S, (**b**) 80S-Zn5, (**c**) 80S-Ag5, and (**d**) 80S-Zn3Ag2.

To better understand the impact of adding zinc and silver on the structure of the glass, the materials 80S, 80S-Zn5, 80S-Ag5, and 80S-Zn3Ag2 were analysed using ^29^Si MAS NMR, and the spectra were calculated using the Gaussian deconvolution method described previously [41]. The chemical shifts (CS), network connectivity (Q), and average network connectivity (Q^n^) of each material are shown on the spectra (Figure 8), and the results of the full width at half maximum (FWHM) and the percentage of the area under curve are listed in Table 1. The three peaks at −110.4, −100.3, and −89.8 ppm in the spectrum of 80S were assigned to the Q^4^, Q^3^, and Q^2^ species, respectively (Figure 8a), and similar patterns were observed for the other three materials. The dominant structural unit of 80S was the Q^4^ species, for which the percentage of area was 57.28%, followed by Q^3^ (41.18%) and Q^2^ (1.54%), with a Q^n^ of 3.56 (Table 1). Compared with 80S, 80S-Zn5 was observed to have slight decreases in the Q^4^ (54.19%) and Q^3^ (39.05%) species and an increase in the Q^2^ (6.76%) species, whereas a decrease in Q^n^ was observed (3.47) (Figure 8b and Table 1). In the 80S-Ag5 material, the Q^4^ (59.21%) peak was dominant and shared a similar value to that of 80S (Figure 8c and Table 1); the Q^3^ (34.18%) peak decreased slightly, and the Q^2^ (6.61%) peak increased, with a similar Q^n^ of 3.52 to that of 80S. Interestingly, the material 80S-Zn3Ag2 showed an observable increase in the Q^4^ (69.88%) peak compared with the others, a decreased Q^3^ (27.32%) peak, and a similar Q^2^ (2.80%) peak to that of 80S (Figure 8d and Table 1), with an increased Q^n^ of 3.61.

### 2.3. Antibacterial Activity of 80S-ZnAg

**Figure 9:**
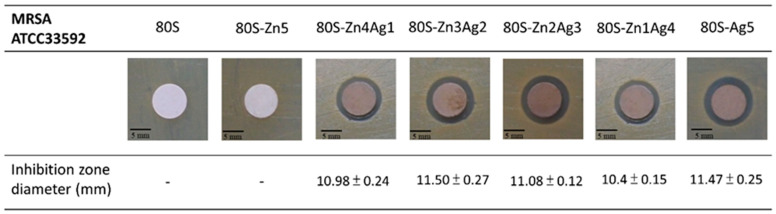
Disk diffusion test of the 80S-ZnAg materials against MRSA ATCC33592. The diameters of the inhibition zones are presented as the mean ± standard deviation.

**Figure 10:**
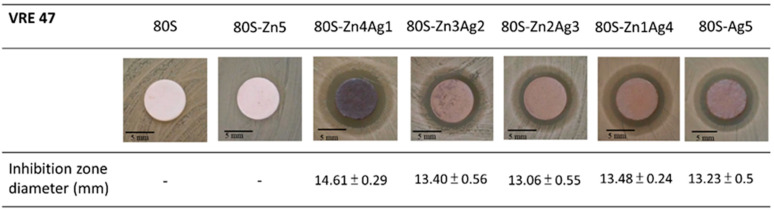
Disk diffusion test of the 80S-ZnAg materials against VRE, clinical strain 47. The diameters of inhibition zones are presented as the mean ± standard deviation.

Since 80S-Zn5 and 80S-Zn10 were observed to have no antibacterial activity against MRSA ATCC 33592 (Figure S3), silver was added to enhance the antibacterial effect of the materials. Inhibition zones were detectable for all 80S-ZnAg materials and 80S-Ag5 against both MRSA ATCC33592 and *E. faecalis* VRE-47 (Figure 9 and Figure 10). In the examination of the antibacterial activity against MRSA ATCC 33592, 80S-Zn3Ag2 presented antibacterial activity similar to that of 80S-Ag5, and it was more robust than the other materials (Figure 9). However, the largest inhibition zone against *E. faecalis* VRE-47 was observed for 80S-Zn4Ag1, followed by 80S-Zn1Ag4, 80S-Zn3Ag2, 80S-Ag5, and 80S-Zn2Ag3 (Figure 10). The difference between the inhibition zones of MRSA and VRE illustrated the better antibacterial effect of 80-ZnAg against VRE than against MRSA, although a detailed investigation should be conducted using a kinetic method. Taken together, the results showed that zinc and silver in the 80S-ZnAg materials might have synergistic antibacterial effects, as 80S-Zn3Ag2 could release zinc and more silver than other 80S-ZnAg materials (Figure 4), and so it was subjected to kinetic antibacterial analysis.

**Figure 11:**
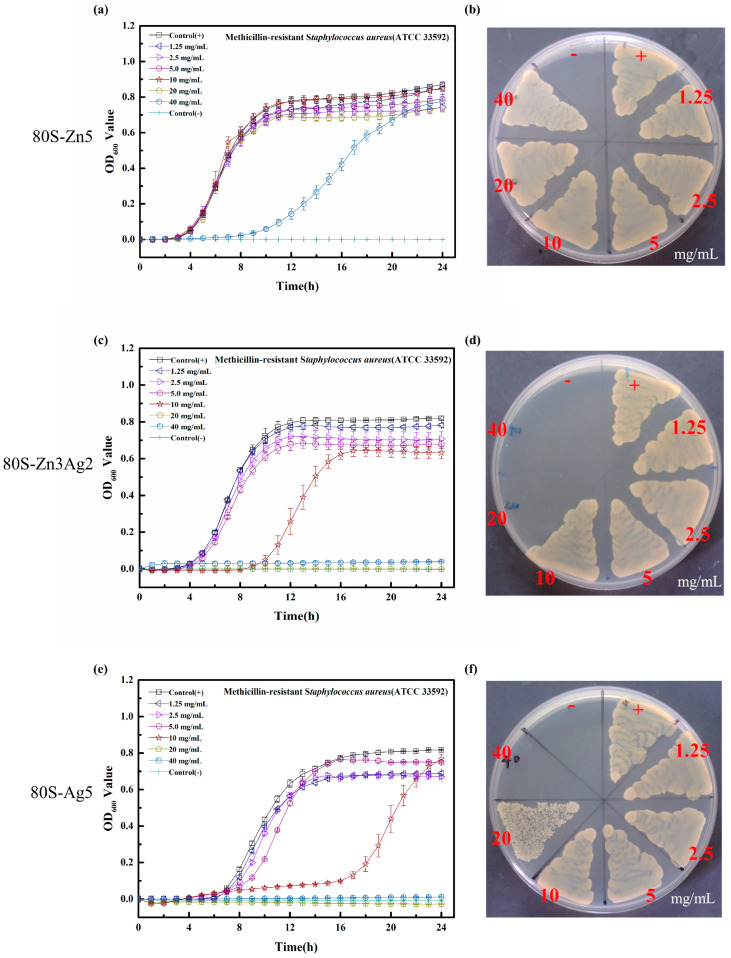
Bacterial growth curve (**a**,**c**,**e**) and the colony-forming test (**b**,**d**,**f**) of 80S-Zn5 (**a**,**b**), 80S-Zn3Ag2 (**c**,**d**), and 80S-Ag5 (**e**,**f**) against MRSA ATCC33592. Dots represent the mean absorbance, and the scale shows the standard deviation of 3 independent experiments. Control (+), positive bacterial growth without treatment; control (-), no bacterial inoculum. The materials were prepared using TSB for the experiments against MRSA.

**Figure 12:**
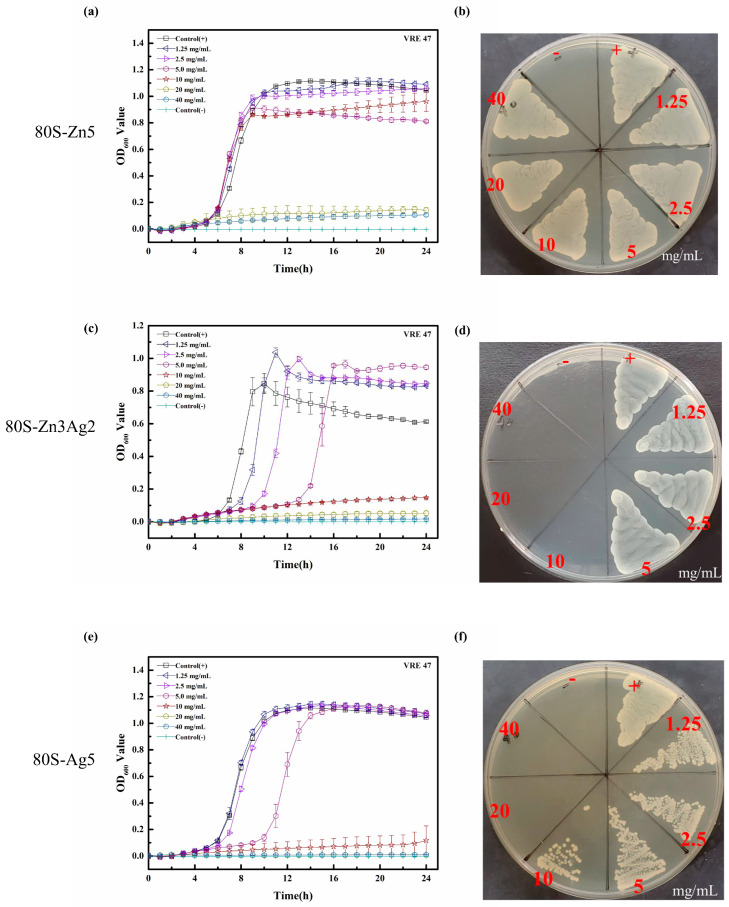
Bacterial growth curve (**a**,**c**,**e**) and the colony-forming test (**b**,**d**,**f**) of 80S-Zn5 (**a**,**b**), 80S-Zn3Ag2 (**c**,**d**), and 80S-Ag5 (**e**,**f**) against VRE, clinical strain 47. Dots represent the mean absorbance, and the scale shows the standard deviation of 3 independent experiments. Control (+), positive bacterial growth without treatment; control (-), no bacterial inoculum. The materials were prepared using BHI for the experiments against VRE.

Although postponed growth was noted in the growth curves of MRSA ATCC 33592 at 40 mg/mL 80S-Zn5 (Figure 11a), it showed no antibacterial effect, similar to that in the disk diffusion assays (Figure 11b). With the addition of silver, 80S-Zn3Ag2 was found to have enhanced antibacterial activity against MRSA ATCC 33592 (Figure 11c), with postponed growth at 10 mg/mL and an MIC of 20 mg/mL. A similar growth curve was observed for 80S-Ag5 (Figure 11e). Notably, the MBC of 80S-Zn3Ag2 against MRSA ATCC 33592 was 20 mg/mL (Figure 11d), which was lower than that of 80S-Ag5 (40 mg/mL) (Figure 11f). Neither an antibacterial effect nor postponed growth was observed for 80S-Zn5 against *E. faecalis* VRE-47 (Figure 12a,b). The delayed growth of *E. faecalis* VRE-47 was noted at 1.25, 2.5, and 5.0 mg/mL 80S-Zn3Ag2, with MIC and MBC values of 10 mg/mL (Figure 12c,d). However, the delayed growth of *E. faecalis* VRE-47 was found only at 5 mg/mL 80S-Ag5, whereas the MIC and MBC were 10 and 20 mg/mL, respectively (Figure 12e,f). Notably, concentrations above the MIC inhibited bacterial cells constantly, with no growth observed in 24 h.

### 2.4. Bioactivity of 80S-ZnAg

**Figure 13:**
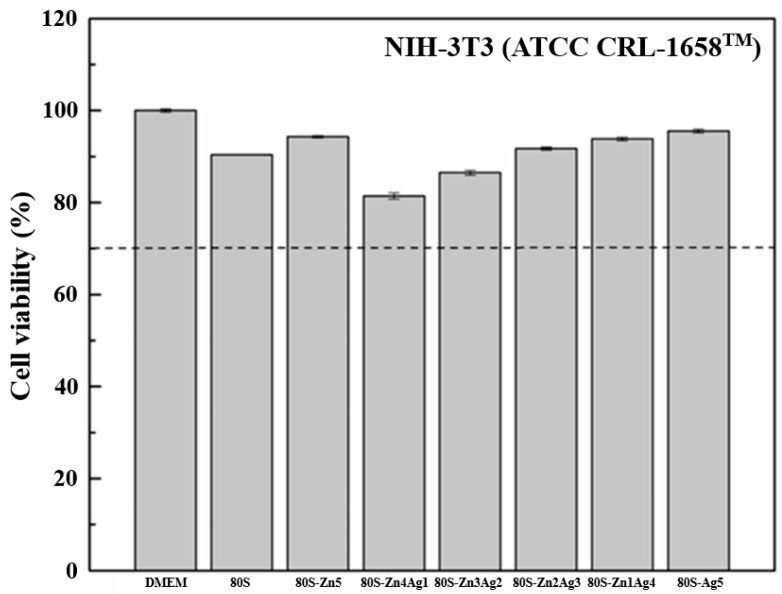
In vitro toxicity test results of DMEM (control group), 80S, 80S-Zn5, 80S-Zn4Ag1, 80S-Zn3Ag2, 80S-Zn2Ag3, 80S-Zn1Ag4, and 80S-Ag5 at 20 mg/mL against the NIH-3T3 cell line. Dashed line, 70% cell viability.

The cytotoxicities of all the materials (80S, 80S-Zn5, 80S-Zn4Ag1, 80S-Zn3Ag2, 80S-Zn2Ag3, 80S-Zn1Ag4, and 80S-Ag5) were evaluated at 20 mg/mL (Figure 13). Compared with the control group (DMEM), the viability of the NIH-3T3 cells treated with the materials was higher than 70% (the dashed line of Figure 13), which was defined as biocompatibility according to the guideline recommended in ISO-10993-5.

**Figure 14:**
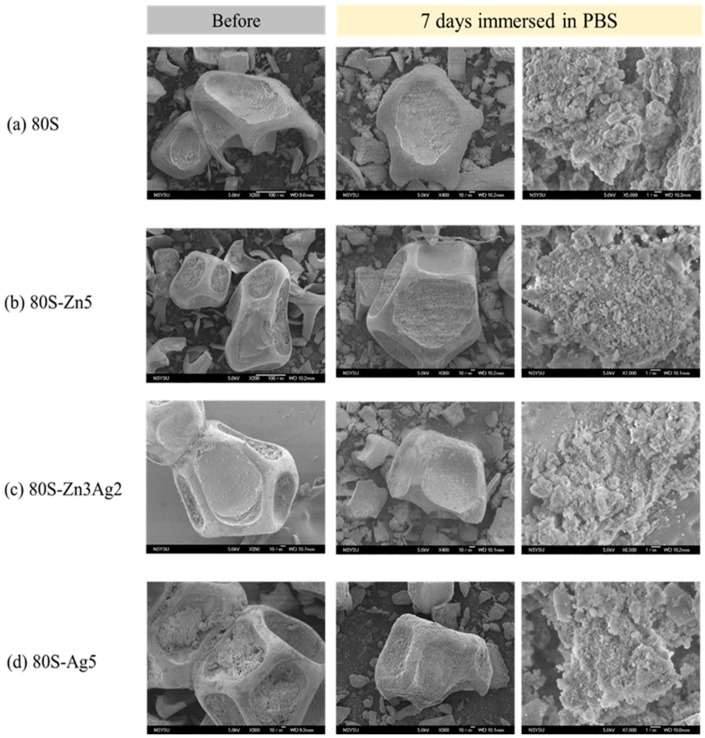
SEM images of the (**a**) 80S, (**b**) 80S-Zn5, (**c**) 80S-Zn3Ag2, and (**d**) 80S-Ag5 materials before and after 7 days of immersion in PBS.

**Figure 15:**
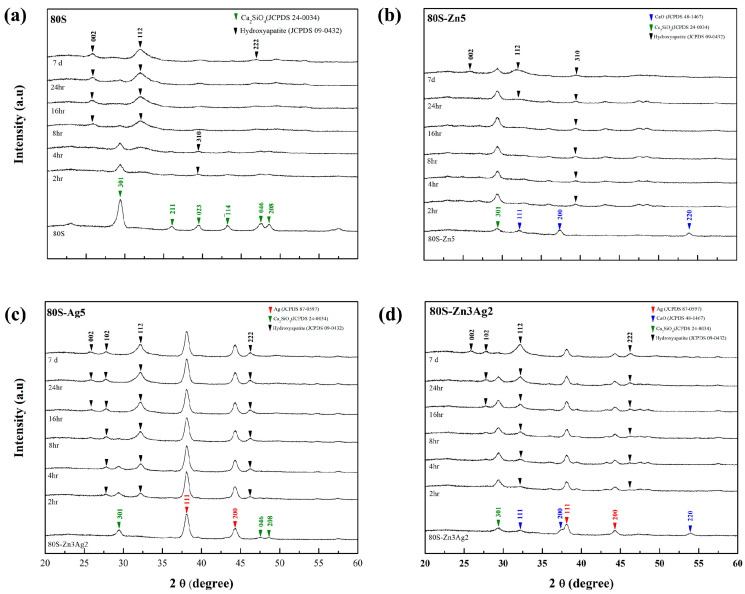
XRD patterns of the HA formed by (**a**) 80S, (**b**) 80S-Zn5, (**c**) 80S-Ag5, and (**d**) 80S-Zn3Ag2 before immersion and after 2 h, 4 h, 8 h, 16 h, 24 h, and 7 days of immersion in PBS.

**Figure 16:**
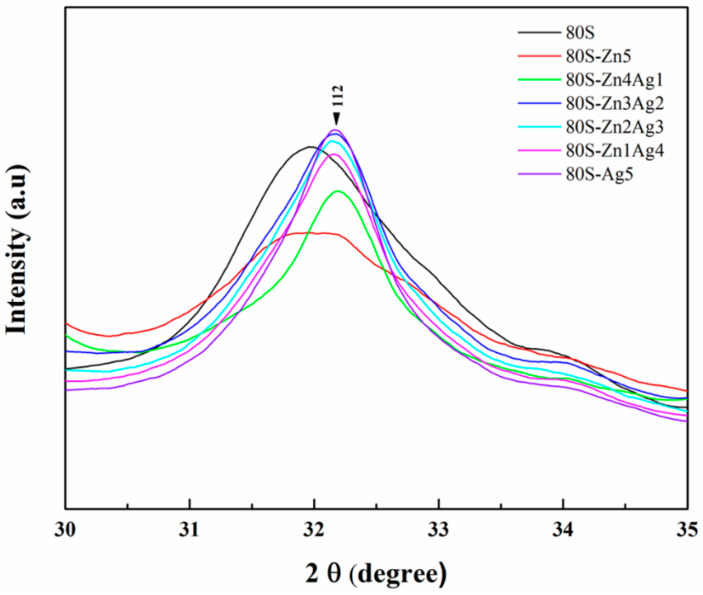
XRD patterns of the 80S-ZnAg materials at the 2θ range from 30° to 35° after 7 days of immersion in PBS.

The 80S-ZnAg materials were immersed in phosphate-buffered saline for different time periods, and then micrographs were captured using SEM (Figure 14). Needle-like crystals were observed on the surfaces of the materials (80S, 80S-Zn5, 80S-Zn3Ag2, and 80S-Ag5) after 7 days of immersion, suggesting that they had the ability to form HA. To assess the abilities of all the materials to form HA, XRD analyses were carried out to record the lattice planes of the materials after 2 h, 4 h, 8 h, 16 h, 24 h, and 7 days of immersion (Figure 15). Without the addition of silver or zinc, 80S showed the rapid formation of HA after 2 h of immersion (Figure 15a), with a lattice plane of (310) at 2θ = 39.58° (JCPDS No. 09-0432, HA). Immersion of 80S for 24 h generated (002) and (112) planes at 2θ = 25.82° and 32.20°, and a (222) plane at 2θ = 46.92° was observed after 7 days. Similar situations were noted for 80S-Zn5 (Figure 15b) and 80S-Ag5 (Figure 15c), whereas 80S-Zn5 exhibited a weaker formation of HA after 7 days than 80S and 80S-Ag5, with an obviously lower peak of the (112) plane at 2θ = 32.20°. Remarkably, 80S-Zn3Ag2 was found to have a similar ability to form HA as 80S and 80S-Ag5 (Figure 15d). The 2θ range from 30° to 35° was scanned (0.2°/min) for each material to better understand the intensity of the (112) plane at 2θ = 32.20° (Figure 16). The highest peak was noted for 80S-Ag5, followed by 80S-Zn3Ag2, 80S-Zn2Ag3, 80S, 80S-Zn1Ag4, 80S-Zn4Ag1, and 80S-Zn5.

## 3. Discussion

In this study, we sought to synthesize a zinc and silver codoped bioactive material. The TGA observations agreed with previous studies where similar glass systems were used [23,42,43], implying the purity of our materials. Furthermore, the changes in the XRD patterns suggested that zinc could replace calcium as a network modifier due to zinc having a lower ionic radius and a higher bonding strength than calcium [44]. In this scenario, calcium formed CaO on the surface of the materials and could be detected. Ten molar ratios of zinc were added during the preparation, and 80S-Zn10 was generated. As shown in Figure S4, the overdosage of zinc formed ZnO on the surface of the material, with (002) and (102) planes at 2θ = 34.42° and 47.53°, respectively (JCPDS No. 36-1451). Our XRD results agreed with the previous efforts reported by Sergi et al. and Chen et al. [26,45] and proved that zinc and silver were coincorporated in the 80S system. The UV-vis result of 80S-Zn5 demonstrated a peak at 350 nm, which indicated the absorbance of ZnO. The absorbance peak in the UV-vis spectrum might have been affected by the particle sizes or the composition of the additions [46]. In this study, with an increased molar ratio of Zn, 80S-Zn10 showed a much higher signal at 344 nm than 80S-Zn5 (Figure S5), implying that zinc was incorporated in the 80S system in the form of ZnO [47,48].

According to Dietzel’s field strength criterion [49], cation ions can be judged as network formers, network modifiers, and/or intermediate ions. When the field strength was strong (1.3–2.0), the ions were regarded as network formers, such as Si, B, and P; conversely, when the field strength was weak (0.1~0.4), the ions were classified as network modifiers, including Na, Ca, and K. The intermediate ions, such as Zn, Ga, and Ce, had field strengths ranging from 0.4 to 1.3 but could not form a glass structure without a network former. In this study, the field strengths of zinc and silver were 0.53 and 0.48, respectively, and both were intermediate ions. Accordingly, the results of the ^29^Si MAS NMR are illustrated in Figure S6. Consistent with the previous finding of Sánchez-Salcedo [42], it was speculated that some of the zinc in 80S-Zn5 was utilized as the network modifier, and thus the Q^n^ decreased from 3.56 to 3.47 compared with that of 80S (Figure 8a,b). However, in this study, zinc could play a role as a network former in 80S-Zn3Ag2, where the zinc molar ratio was less than that of 80S-Zn5, and the Q^n^ increased from 3.56 to 3.67 compared with that of 80S (Figure 8d). In a previous study [50], with the addition of silver, zinc was found to mostly adhere to the surface of the glass, whereas a few ions would become network modifiers, leading to the increased Q^2^ peak (Figure 8c).

ZnO was previously reported to possess antibacterial activities [51], and its mechanism was attributed to the inhibition of growth and the induction of ROS by Zn^2+^ [52,53]. The synergistic antimicrobial effect of silver and zinc was previously examined against *E. coli* ATCC 11229 and *B. subtilis* ATCC 23857 using individual salts [37]. Bednář et al. synthesized a silicate net nanostructure with zinc, and silver nitrate was then doped into the structure using a photocatalytic reduction method [38]. Although remarkable antibacterial activities were observed, only drug-sensitive bacteria were examined in their work. In another study, a senary SiO_2_-P_2_O_5_-CaO-SrO-Ag_2_O-ZnO glass system was prepared via the sol-gel method, designated BG-AZ [39]. However, low antibacterial activities were noted for BG-AZ, and extremely low zinc release (0.06–0.16 ppm) was also noted. In our study, silver and zinc were coreleased from the glass structure, and kinetic antibacterial analyses revealed the synergistic effect of the coreleased silver and zinc against drug-resistant bacteria (Figure 11 and Figure 12), where 80S-Zn3Ag2 demonstrated more marked antibacterial activity than the others. The findings supported the aforementioned hypothesis of the synergetic antibacterial effect of silver and zinc.

Previous efforts have documented that bioactive glass is capable of forming hydroxyapatite (HA) on the surface, and the characteristics might be influenced by the addition of metal [23,24,25,26]. In this study, due to the addition of silver, lattice distortion and a peak shift were noted in the silver-containing materials, which agreed with a previous study [54]. It was speculated that in 80S-Zn5, ZnO_4_^2−^ would need Ca^2+^ for balancing the charge [24], and/or Zn^2+^ would influence the ion exchange between Ca^2+^ and H^+^/H_3_O^+^ [55], leading to low HA formation.

## 4. Materials and Methods

### 4.1. Preparation of 80S-ZnAg

Zinc and silver containing 80S, a SiO_2_-CaO-P_2_O_5_ system with a molarity ratio of 80:15:5, were synthesized using a sol-gel method as described in a previous study, with some modifications [56,57]. Zinc and/or silver were applied at ratios of 5*x* and *x*, respectively, in which *x* could be 0, 1, 2, 3, 4, and 5. The products were designated as 80S-ZnAg, including 80S-Zn5, 80S-Zn4Ag1, 80S-Zn3Ag2, 80S-Zn2Ag3, 80S-Zn1Ag4, and 80S-Ag5. Briefly, the structure-directing agent, pluronic F127, was added to 2 M HNO_3_ in absolute ethanol and mixed with tetraethylorthosilicate (TEOS), triethyl phosphate (TEP), calcium nitrate (Ca(NO_3_)_2_⋅4H_2_O), and different molar ratios of zinc nitrate (ZnNO_3_) and silver nitrate (AgNO_3_). The mixture was then stirred at 25 °C for 24 h to form the sol. The template, polyurethane foam (PUF), was soaked into the sol twice for gelation and was then aged at 25 °C for 3 days. Subsequently, the gel was subjected to thermal treatment with a heating rate of 2.5 °C/min and maintenance at 700 °C for 3 h to remove the precursors, such as F127, PUF, and acids. The resulting products were collected for further examination.

### 4.2. Characterization of 80S-ZnAg

The 80S-ZnAg materials were subjected to thermogravimetric analysis (TGA), X-ray diffraction (XRD), and ultraviolet-visible (UV-vis) spectroscopy. TGA was conducted using thermal analysers (Mettler-Toledo, 2-HT). The samples were heated from room temperature to 1000 °C with an airflow of 35 mL (STP)/min and a heating rate of 10 °C/min. The structure and crystallinity of 80S-ZnAg were analysed using a LabX XRD-6000 diffractometer (Shimadzu, Kyoto, Japan) at 40 mA and 30 kV with Cu Kα radiation (λ = 1.542 Å) and in the 2θ range from 20° to 80° (5°/min). The results were annotated in accordance with the database set up by the Joint Committee on Powder Diffraction Standards (JCPDS). The UV-vis spectra of 80S-ZnAg from 200 to 800 nm were recorded using a Shimadzu UV-2600 spectrophotometer (Shimadzu, Japan).

### 4.3. Inductively Coupled Plasma-Mass Spectrometry (ICP-MS)

All 80S-ZnAg materials (80S-Zn5, 80S-Zn4Ag1, 80S-Zn3Ag2, 80S-Zn2Ag3, 80S-Zn1Ag4, and 80S-Ag5) were soaked in tryptic soy broth (TSB) as described in a previous study [57]. In brief, 40 mg of 80S-ZnAg powder was soaked in 1 mL of tryptic soy broth (TSB), and the solution was shaken at 200 rpm for 24 h. After soaking, the supernatant was obtained by centrifugation at 3000 rpm and filtered through a 0.22 μm filter to remove impurities, yielding the 80S-ZnAg. The silver and zinc contents of the 80S-ZnAg were determined using an inductively coupled plasma-mass spectrometer (Thermo-Element XR).

### 4.4. Solid-State Magic Angle Spinning Nuclear Magnetic Resonance (^29^Si MAS NMR)

Solid-state ^29^Si MAS NMR of 80S-ZnAg was conducted using a Bruker Avance III 400 spectrometer with a spinning speed of 10 KHz for all experiments. The conditions included a pulse width of 1.1 μs, a relaxation delay of 10 s, a magnetic field strength of 9.39 Tesla, scanning numbers of 4600, and an acquisition time of 0.012 s. Tetramethyl-silicane was used as a reference.

### 4.5. Morphology of 80S-ZnAg

The selected materials (80S, 80S-Zn5, 80S-Zn3Ag2, and 80S-Ag5) were transferred onto carbon tape and mounted on scanning electron microscope (SEM) sample holders. The holders with the samples were dried at 60 °C for 3–5 days and were coated with gold in an ion coater. Micrographs of the materials’ surfaces were captured using a scanning electron microscope (JSM-6330TF, JEOL, Tokyo, Japan).

The materials were mixed with distilled water and spread onto copper transmission electron microscope (TEM) grids, and the sample holders were dried at 60 °C for 3–5 days. A transmission electron microscope (Tecnai F20 G2 MAT S-TWIN) was used to record the morphology of the materials. High-angle annular darkfield scanning TEM (HAADF-STEM) tomography was also performed with an energy-dispersive X-ray spectrometer (EDS).

### 4.6. Bacterial Strains

Two drug-resistant bacterial strains were included in this study: methicillin-resistant *S. aureus* (MRSA) ATCC 33592 and vancomycin-resistant *E. faecalis* VRE-47. The bacteria were stored at −80 °C with 15–25% glycerol until testing. The testing strains were recovered on an agar plate containing 5% sheep blood (Creative Media Plate, New Taipei City, Taiwan) or a brain–heart infusion (BHI) agar plate at 37 °C for 16–18 h. To stabilize the bacteria’s physiological characteristics, the recovered bacteria were cultivated onto a fresh agar plate and incubated at 37 °C for 16–18 h.

### 4.7. Disk Diffusion

Powders of the 80S-ZnAg materials (80S-Zn5, 80S-Zn4Ag1, 80S-Zn3Ag2, 80S-Zn2Ag3, 80S-Zn1Ag4, and 80S-Ag5) were compressed into 8 mm disks for testing. Bacterial colonies were scraped into phosphate-buffered saline (PBS) and adjusted to a concentration of 2 × 10^8^ CFU/mL. The adjusted suspensions were spread onto TSB agar in the case of MRSA ATCC 33592 or onto BHI agar in the case of *E. faecalis* VRE-47. Next, the material disks were carefully placed on the plates using sterile forceps. The plates were incubated at 37 °C for 16–18 h, and photos of the results were taken to measure the diameter of the inhibition zone.

### 4.8. In Vitro Kinetic and Colony-Forming Assays

The bacterial growth of MRSA ATCC 33592 or *E. faecalis* VRE-47 was recorded under various treatments of the materials (80S-Zn5, 80S-Zn3Ag2, and 80S-Ag5), as previously described [50]. In short, the materials were prepared in TSB or BHI at 80 mg/mL, as mentioned above, and the solutions were diluted in a twofold series, resulting in concentrations between 2.5 and 80 mg/mL. The bacterial suspension for testing was prepared at a concentration of 2 × 10^8^ CFU/mL and diluted 200-fold. Subsequently, 100 μL of the solutions at various concentrations was mixed with 100 μL of the diluted bacterial suspension in a 96-well plate, with a final bacterial density of 5 × 10^5^ CFU/mL and with the concentrations of the solution ranging from 1.25 to 40 mg/mL. The 96-well plate was incubated at 37 °C, and the absorbance was recorded hourly over 24 h using a spectrophotometer (Tecan, Zurich, Switzerland). The minimum inhibitory concentration (MIC) was interpreted as the minimum concentration without visible growth.

After 24 h of incubation, 1 μL of the bacterial cultures in each well was inoculated onto TSB or BHI agar using a standard 1 μL loop (BD, Difco ^TM^, Cat. No. 220215). The plates were incubated at 37 °C for 16–18 h. The images of the results were recorded, and the lowest concentration without bacterial colonies was defined as the minimum bactericidal concentration.

### 4.9. Bioactivity of 80S-ZnAg

The cytotoxicity assays were carried out in accordance with the guidelines recommended by the American National Standard ISO 10993-5 [58]. In total, 10^4^ NIH-3T3 cells were first cultured for 1 day and incubated with 20 mg/mL of the materials at 37 °C with 5% CO_2_ for another day. Dulbecco’s modified Eagle medium was utilized as a control. After incubation, the medium was removed from the wells, and the XTT cell proliferation assay kit (Biological Industries, Kibbutz Beit Haemek, Israel) was used to measure the viability. The absorbances at 492 nm were recorded using an ELISA reader (SPECTROstar Nano, BMG LABTECH, Offenburg, Germany).

For the hydroxyapatite (HA) formation assays, the 80S-ZnAg materials were immersed in PBS for 2 h, 4 h, 8 h, 16 h, 24 h, and 7 days. After immersion, the samples were dried and subjected to XRD and SEM analyses.

## 5. Conclusions

Taken together, our findings revealed the chemical changes in the 80S-ZnAg materials synthesized using a sol-gel method. Among the 80S-ZnAg materials, 80S-Zn3Ag2 had the most remarkable and synergistic antibacterial activities against antibiotic-resistant crises, including MRSA and VRE. Additionally, 80S-Zn3Ag2 demonstrated low cytotoxicity and biocompatibility and a high capability to form HA. Further studies on its clinical application are needed to develop an innovative bone/dental material to address the issue of antibiotic resistance.

## Figures and Tables

**Table 1:** Chemical shifts and relative peak areas obtained by solid-state ^29^Si MAS NMR spectroscopy.

	Q^4^			Q^3^			Q^2^			<Q^n^>
	CS, ppm	Area (%)	FWHM, ppm	CS, ppm	Area (%)	FWHM, ppm	CS, ppm	Area (%)	FWHM, ppm	
80S	−110.40	57.28	8.64	−100.30	41.18	6.68	−89.84	1.54	3.05	3.56
80S-Zn5	−109.90	54.19	8.97	−99.58	39.05	8.65	−90.21	6.76	3.61	3.47
80S-Ag5	−109.90	59.21	8.49	−100.05	34.18	7.26	−90.94	6.61	6.30	3.52
80S-Zn3Ag2	−110.00	69.88	8.97	−99.76	27.32	8.65	−90.19	2.80	3.61	3.67

## Data Availability

Data are available when requested from authors.

## Supplementary Materials

The following supporting information can be downloaded at https://www.mdpi.com/article/10.3390/ijms241813679/s1.
