# Supplementary material for: Synthesis, Characterization, and Bioactivity of Mesoporous Bioactive Glass Codoped with Zinc and Silver
Source: Int J Mol Sci. 2023 Sep 5;24(18):13679. doi: 10.3390/ijms241813679 (PMC10531463; doi:10.3390/ijms241813679)
Supplement: Supplementary file 1 [file ijms-24-13679-s001.zip › ijms-2587832-supplementary.pdf]

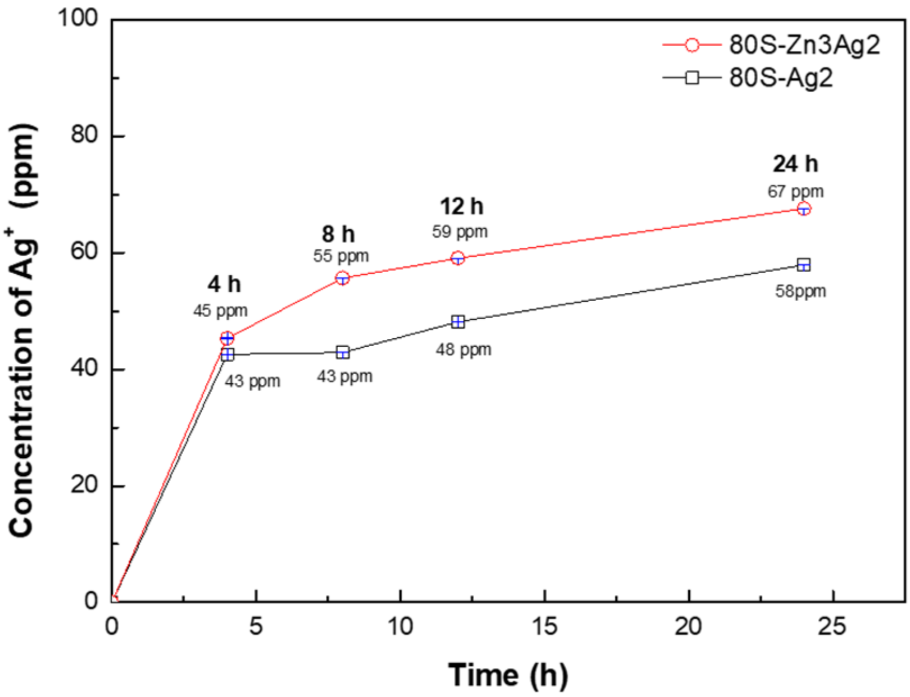

Figure S1. ICP-MS results of time-dependent silver ions released test of 80S-Ag2 and 80S-Zn3Ag2.

(a) 80S-Zn5

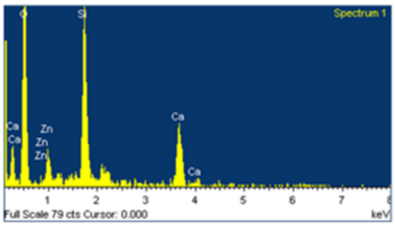

Element	Weight%	Atomic%
O K	52.59	71.21
Si K	19.90	15.35
Ca K	20. 69	11.18
Zn L	6.82	2.26
Totals	100.00	

(b) 80S-Zn3Ag2

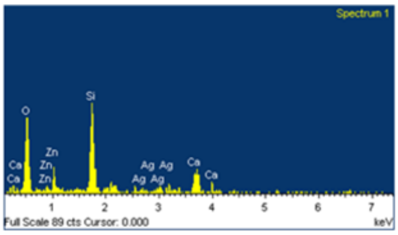

Element	Weight%	Atomic%
O K	39.02	61.27
Si K	23.12	20.68
Ca K	20.11	12.61
Zn L	8.61	3.31
Ag L	9.14	2.13
Totals	100.00	

(c) 80S-Ag5

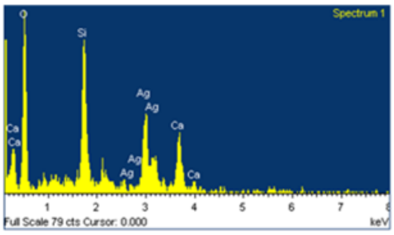

Element	Weight%	Atomic%
O K	43.63	72.56
Si K	11.34	10.75
Ca K	13.39	8.89
Ag L	31.64	7.80
Totals	100.00	

Figure S2. EDS results of (a)80S-Zn5, (b)80S-Zn3Ag2 and (c)80S-Ag5.

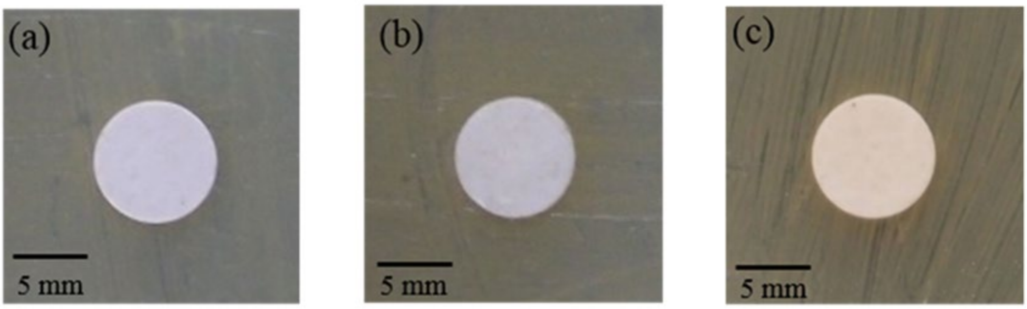

Figure S3. Antibacterial activity against MRSA (ATCC 33592) results of (a)80S, (b)80S-Zn5, and (c) 80S-Zn10.

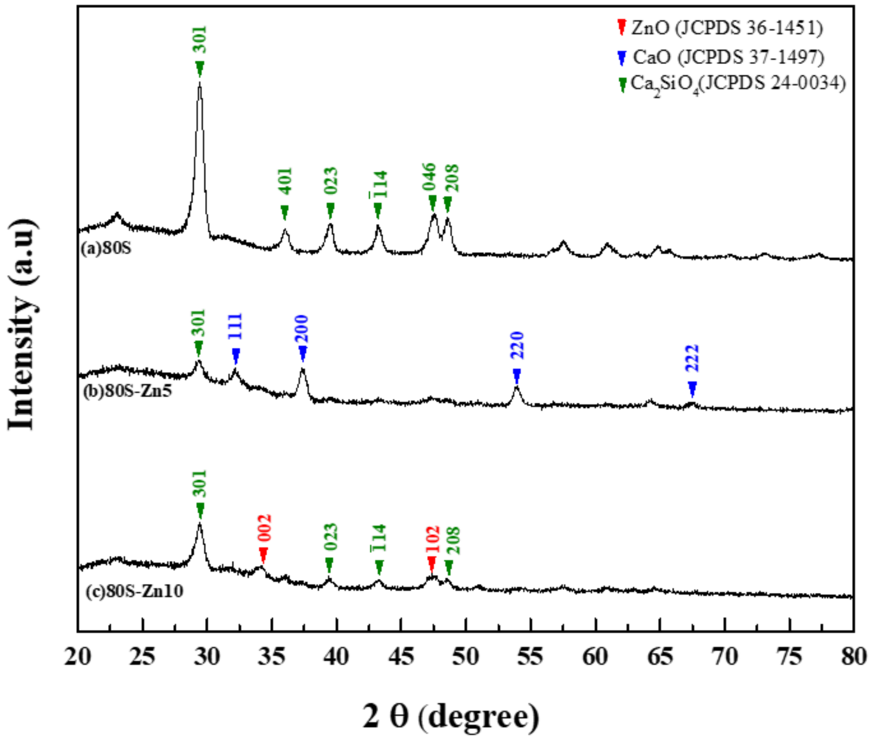

Figure S4. XRD patterns of (a) 80S, (b) 80S-Zn5, and (c) 80S-Zn10.

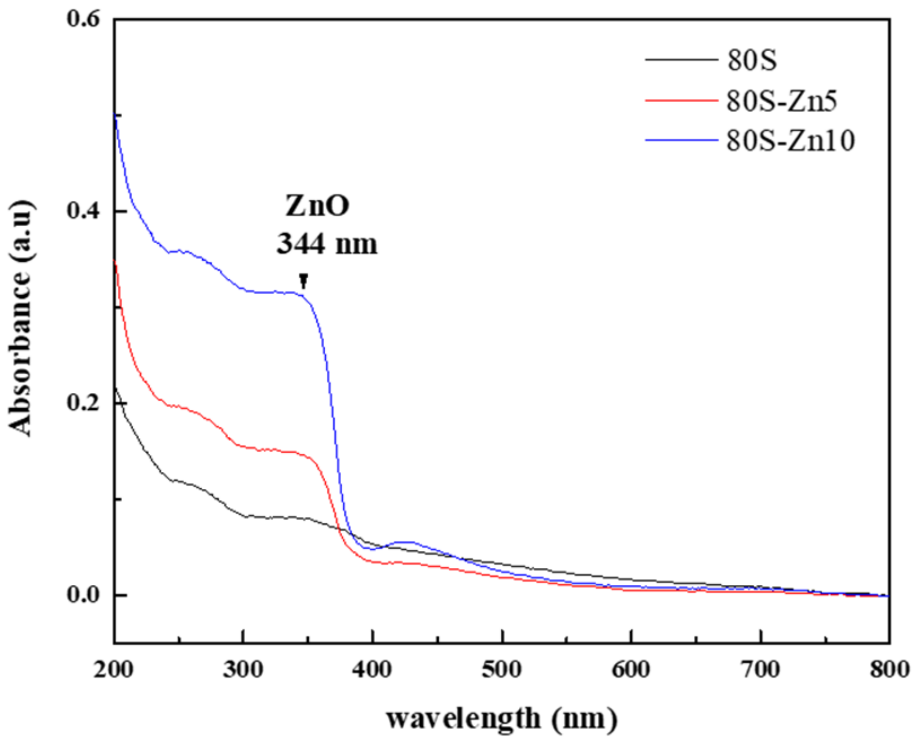

Figure S5. UV-vis results of 80S, 80S-Zn5, and 80S-Zn10.

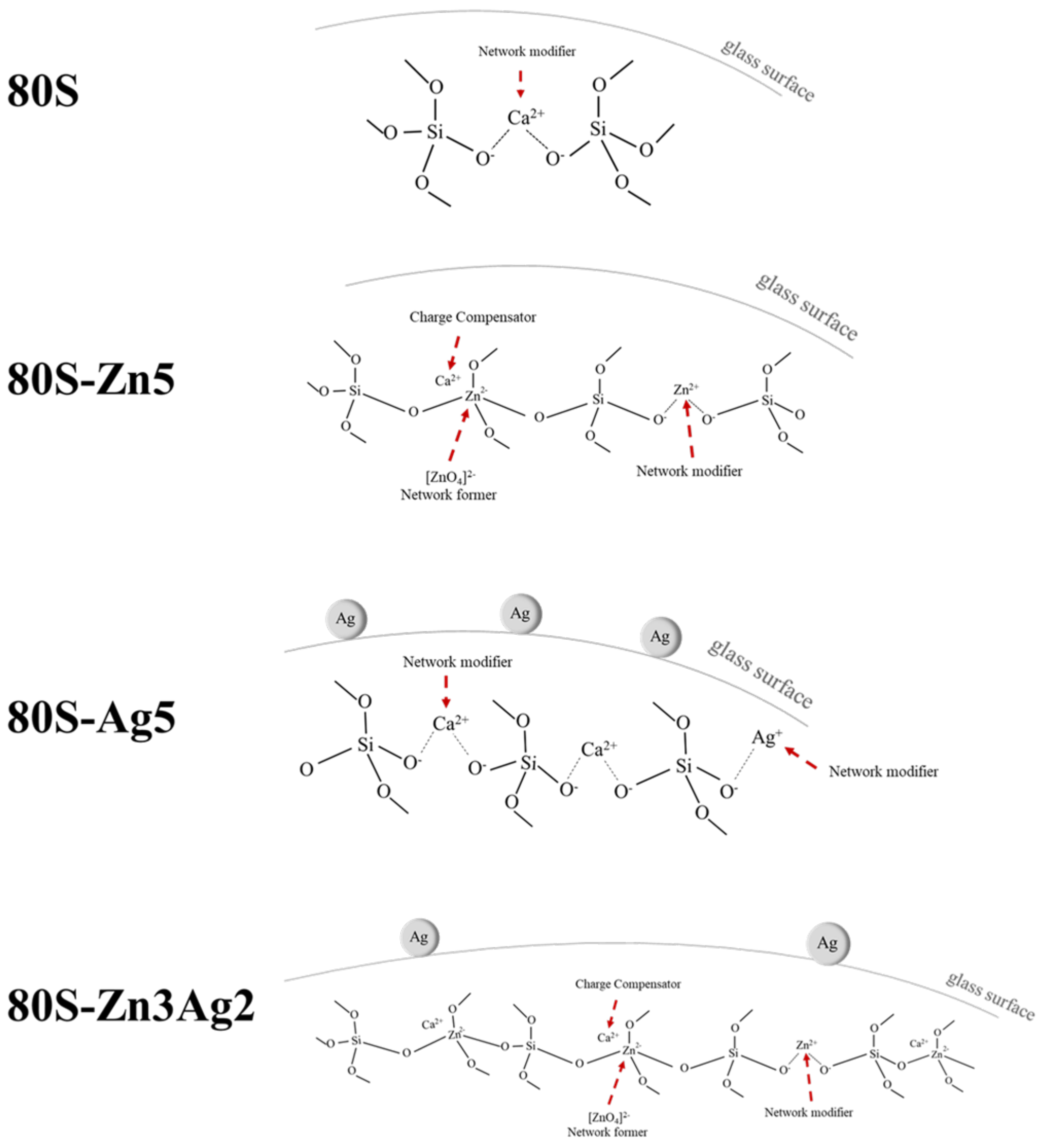

Figure S6. The glass structures were speculated using solid-state ^{29}Si MAS NMR results of (a)80S, (b)80S-Zn5, (c)80S-Ag5, and (d)80S-Zn3Ag2.
